# The NLRP3 inflammasome in metabolic dysfunction-associated steatotic liver disease: role and advances in therapeutic targeting

**DOI:** 10.3389/fimmu.2026.1924392

**Published:** 2026-08-03

**Authors:** Sheng Wei, Yanlang He, Chen Luo, Xiaofeng He

**Affiliations:** 1 Department of General Medicine, The Second Affiliated Hospital of Wannan Medical College, Wuhu, China; 2 Department of Infectious Disease, Shaoyang Central Hospital, Shaoyang, China

**Keywords:** liver fibrosis, metabolic dysfunction-associated steatotic liver disease (MASLD), NLRP3 inflammasome, pyroptosis, therapeutic target

## Abstract

Metabolic dysfunction-associated steatotic liver disease (MASLD), formerly termed non-alcoholic fatty liver disease (NAFLD), has become the most prevalent chronic liver disease worldwide. Its disease spectrum begins with simple steatosis, progresses through steatohepatitis (MASH, formerly NASH) and liver fibrosis, and can eventually advance to cirrhosis and even hepatocellular carcinoma. With deeper insight into its metabolic nature, the nomenclature of this disease has been updated from NAFLD to MASLD. During the transition from simple lipid accumulation to necroinflammation, excessive activation of innate immunity constitutes a decisive step, and the NLRP3 inflammasome serves as a central hub linking hepatic metabolic stress to sterile inflammation. The NLRP3 inflammasome is assembled from the sensor protein NLRP3, the adaptor protein ASC, and the effector protein pro-caspase-1, and is triggered by a two-signal model comprising priming and activation. In the metabolic microenvironment of MASLD, multiple danger signals—including free fatty acids, cholesterol crystals, reactive oxygen species, molecules released from damaged mitochondria, endoplasmic reticulum stress, and gut-derived lipopolysaccharide—can drive its activation in hepatocytes, Kupffer cells, hepatic stellate cells, and other cell types. Subsequently, caspase-1 catalyzes the maturation of IL-1β and IL-18 and cleaves gasdermin D to mediate hepatocyte pyroptosis, thereby amplifying liver inflammation, promoting stellate cell activation and extracellular matrix deposition, and contributing to inflammation-associated hepatocellular carcinoma. Given its pivotal position in this pathological network, targeting the NLRP3 inflammasome is considered a promising strategy to simultaneously curb inflammation and fibrosis. This narrative review examines the structure and activation mechanisms of the NLRP3 inflammasome and its roles in each stage of MASLD progression, summarizes current research on multi-level intervention strategies—represented by synthetic inhibitors, natural products, traditional Chinese medicine, and metabolic regulation—and analyzes current deficiencies in clinical translation, thereby providing a reference for future mechanistic studies and drug development.

## 1 Introduction

### 1.1 Epidemiological status and disease burden of MASLD

Against the backdrop of the growing epidemics of obesity, type 2 diabetes mellitus, and metabolic syndrome—with a particularly high prevalence of NAFLD/NASH in the type 2 diabetes population (1)—MASLD has overtaken viral hepatitis as the most common chronic liver disease worldwide (2). A systematic review and meta-analysis encompassing 92 studies and over 9 million individuals showed that the global overall prevalence of NAFLD was approximately 30% between 1990 and 2019, with a sustained upward trend, and exceeded 33% in some Asian regions (3). Another meta-analysis of more than 1.2 million individuals estimated the global pooled prevalence at approximately 32%, with a significantly higher prevalence in males than in females (4). Earlier meta-analyses similarly estimated the global prevalence at roughly one-quarter of the population and predicted that the clinical and economic burden would further increase with the spread of metabolic diseases (5, 6). In population-based studies using ultrasound diagnosis, the pooled estimated prevalence also remained stable at approximately 30% (7). The prevalence of NAFLD is even higher in individuals with type 2 diabetes (1); until recently, approved disease-specific pharmacotherapies were lacking, and clinical management relied mainly on lifestyle intervention and comprehensive control of metabolic risk factors (8); this has now begun to change, with the first agents approved for noncirrhotic MASH with moderate-to-advanced fibrosis (see Section 5.5.1). It should be emphasized that MASLD is not a liver-confined disease but a multisystem metabolic disorder (9): cardiovascular disease is the leading cause of death in these patients (10, 11), and MASLD is also associated with an increased risk of extrahepatic complications such as chronic kidney disease (12), which aligns with the systemic low-grade inflammation mediated by NLRP3. MASLD is not a single benign condition but a dynamically evolving disease spectrum: it begins with simple steatosis characterized by pure lipid accumulation in hepatocytes, from which a proportion of patients progress to MASH, marked by hepatocyte injury and lobular inflammation, followed by progressive liver fibrosis, and ultimately advancing to cirrhosis and hepatocellular carcinoma. The transition from simple steatosis to MASH represents a critical juncture for prognostic reversal across the disease spectrum and is the focal point of current mechanistic research and therapeutic intervention.

### 1.2 From NAFLD to MASLD: evolving understanding behind the nomenclature change

For a long time, the diagnosis of NAFLD has relied on the exclusion of other etiologies such as alcohol consumption; this exclusion-based definition poorly reflects the metabolic nature of the disease, and the term “fatty” is considered to carry a certain stigma. In 2020, an international expert panel proposed replacing NAFLD with “metabolic-associated fatty liver disease” (MAFLD), advocating metabolic dysfunction as the core diagnostic criterion (13, 14). In 2023, a consensus led by multiple international liver societies and achieved through a modified Delphi process formally proposed replacing NAFLD with “metabolic dysfunction-associated steatotic liver disease” (MASLD), requiring at least one cardiometabolic risk factor for diagnosis, and introducing an intermediate category, MetALD, for individuals with both metabolic dysfunction and higher alcohol intake (15). This change shifted the disease definition from an exclusion-based approach to one centered on metabolic dysregulation, emphasizing the central role of insulin resistance and associated metabolic abnormalities in pathogenesis. Epidemiological data suggest that the vast majority of patients with former NAFLD meet the new MASLD criteria, and the populations covered by the two nomenclatures overlap substantially. Given that the vast majority of existing literature still uses NAFLD/NASH, this review retains the original terminology when describing previous studies, while aligning with the connotation of MASLD in general discussions concerning metabolic mechanisms.

### 1.3 The “multiple-hit” hypothesis of MASLD pathogenesis

The understanding of MASLD pathogenesis has evolved from the “two-hit” model to the “multiple parallel hits” hypothesis. The latter posits that multiple factors—including insulin resistance, lipotoxicity, oxidative stress, endoplasmic reticulum stress, adipose tissue-derived proinflammatory cytokines, and gut microbiota dysbiosis—act not in a sequential order, but in an intertwined and parallel manner, collectively driving the transition from simple lipid accumulation to necroinflammation in the liver (16–18). Furthermore, genetic susceptibility (e.g., PNPLA3 I148M, TM6SF2 E167K, MBOAT7 rs641738 variants) significantly influences the risk of hepatic steatosis and disease progression (19–21), further highlighting the multifactorial nature of MASLD pathogenesis. In addition, a considerable proportion of patients have lean/non-obese MASLD, and their long-term adverse hepatic outcomes are not better than those of obese individuals, suggesting that diagnosis and management should not be driven solely by body mass index (22, 23). Within this framework, inflammation is no longer viewed as a mere consequence of steatosis but as an active driving force that determines disease progression. The question thus becomes: through what molecular mechanisms does the liver “sense” these heterogeneous metabolic danger signals and convert them into a unified inflammatory output?

### 1.4 The NLRP3 inflammasome: a hub linking metabolic stress to hepatic inflammation

The discovery of the inflammasome has provided a critical clue to the above question. As a cytosolic pattern recognition platform of the innate immune system, the NLRP3 inflammasome can sense a wide array of pathogen-associated molecular patterns and damage-associated molecular patterns, converging them into the activation of caspase-1 and the maturation and secretion of IL-1β and IL-18 (24). In the hepatic microenvironment of MASLD, lipotoxic products, cholesterol crystals, reactive oxygen species, damaged mitochondrial products, and gut-derived endotoxins precisely constitute multiple activation signals for NLRP3, making it a central node that integrates metabolic stress and sterile inflammation. For this reason, the NLRP3 inflammasome has received sustained attention in MASLD research and is regarded as a key target with both mechanistic significance and therapeutic potential (25). As a core node bridging innate immune recognition and metabolic control, the NLRP3 inflammasome is considered to occupy a hub position in the development and progression of metabolic diseases (26, 27); the roles of inflammasomes in various liver diseases have also been systematically reviewed (28). This review focuses on the NLRP3 inflammasome: we first introduce its structure, activation and regulatory mechanisms, then describe its roles in each stage of MASLD from simple steatosis to hepatocellular carcinoma, followed by a summary of multi-level therapeutic strategies targeting NLRP3, and finally discuss the challenges and future directions in this field. This article is a narrative (non-systematic) review. Relevant literature was identified by searching PubMed, Web of Science, and Scopus through 2025 using combinations of the terms “NLRP3”, “inflammasome”, “pyroptosis”, “NAFLD/MASLD”, “NASH/MASH”, “liver fibrosis”, and “hepatocellular carcinoma”, supplemented by manual screening of the reference lists of key articles; representative and mechanistically informative studies were then selected at the authors’ discretion, and no formal systematic-review or meta-analytic methodology was applied.

## 2 NLRP3 inflammasome: an overview

### 2.1 Discovery and research history of the inflammasome

The concept of the inflammasome was first proposed by Martinon et al. in 2002. While investigating the mechanism of IL-1β maturation, they discovered that the activation of caspase-1 depends on a macromolecular complex composed of an NLR protein, the adaptor protein ASC, and inflammatory caspases, and they named this complex the inflammasome (24). Subsequent work demonstrated that hereditary autoinflammatory diseases such as Muckle-Wells syndrome are caused by gain-of-function mutations in NLRP3, establishing the first direct link between NLRP3 and human disease and solidifying its status as the most extensively studied inflammasome member (29). Thereafter, multiple danger signals were shown to trigger sterile inflammation via NLRP3, making this platform a common hub integrating metabolic, infectious, and tissue injury signals (30–35).

### 2.2 Structure and composition of the NLRP3 inflammasome

**Figure 1:**
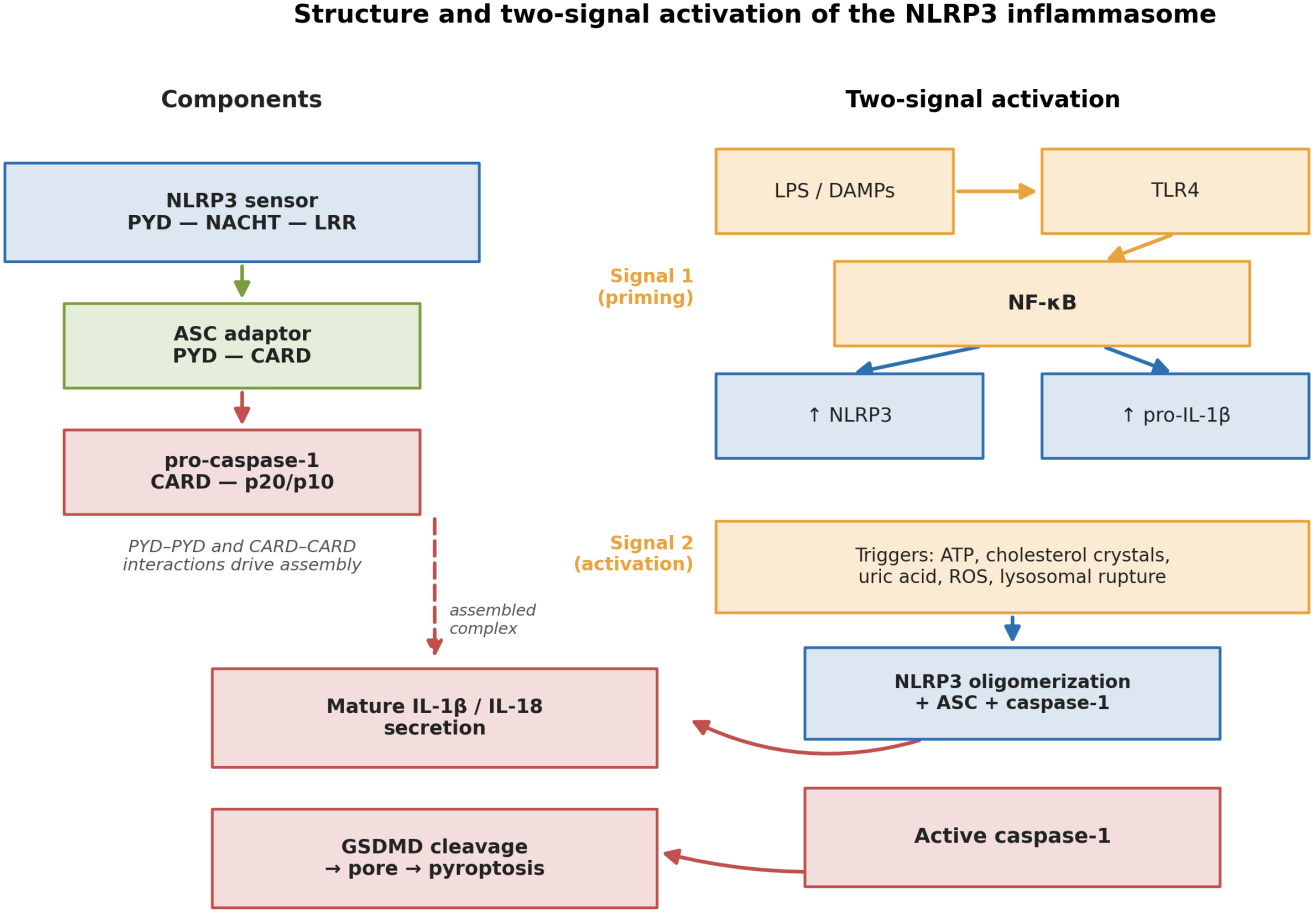
Structure and two-signal activation mechanism of the NLRP3 inflammasome. The sensor NLRP3 (PYD–NACHT–LRR), the adaptor ASC (PYD–CARD), and pro-caspase-1 are shown assembling through PYD–PYD and CARD–CARD interactions. Signal 1 (priming; e.g. TLR4 engagement by LPS) up-regulates NLRP3 and pro-IL-1β via NF-κB, while Signal 2 (activation; K^+^ efflux, mitochondrial ROS, lysosomal rupture, NEK7) drives oligomerization and ASC-speck formation, leading to proximity-induced autoproteolytic activation of caspase-1 and cleavage of pro-IL-1β/pro-IL-18 and GSDMD. Abbreviations are defined in the main text.

The canonical NLRP3 inflammasome consists of three core components. The sensor component, NLRP3, belongs to the NOD-like receptor family and contains an N-terminal pyrin domain (PYD), a central NACHT nucleotide-binding and oligomerization domain, and a C-terminal leucine-rich repeat (LRR) domain. The NACHT domain is responsible for ATP-dependent self-oligomerization, while the LRR domain participates in autoinhibition and signal sensing. The adaptor protein ASC contains both a PYD and a caspase recruitment domain (CARD); it binds to NLRP3 through PYD–PYD interactions and subsequently recruits downstream effector molecules via its CARD. The effector component, pro-caspase-1, is recruited to the complex through CARD–CARD interactions and undergoes proximity-induced autoproteolytic cleavage to become activated. ASC forms filamentous scaffolds via a unified polymerization mechanism involving its PYD and CARD domains, serving as the core of inflammasome assembly (36, 37). The ordered assembly of these three components brings multiple pro-caspase-1 zymogens into close proximity within the ASC scaffold, and it is this proximity-dependent autoproteolytic cleavage of caspase-1—rather than a mere increase in enzymatic output—that converts discrete upstream danger signals into active caspase-1. Recent cryo-electron microscopy studies have further resolved the structural basis of the activated NLRP3 inflammasome disc-shaped complex (38, 39) (**Figure 1**).

### 2.3 Canonical activation mechanism of the NLRP3 inflammasome (two-signal model)

Activation of the NLRP3 inflammasome generally follows a two-signal model, a design that ensures potent pro-inflammatory responses are not triggered inadvertently.

#### 2.3.1 Signal 1 (priming signal)

The first signal, also termed the priming signal, is initiated when pattern recognition receptors such as Toll-like receptors (TLRs) recognize pathogen-associated or damage-associated molecular patterns, leading to the upregulation of NLRP3 itself and pro-IL-1β at the transcriptional level via the NF-κB pathway (40). During this stage, the protein abundance of NLRP3 is elevated from a limiting basal level to a threshold permissive for activation; thus, the priming signal determines the cellular competence to respond to subsequent activation signals. In NAFLD, gut-derived lipopolysaccharide entering the liver through the portal vein and activating TLR4 serves as an important source of this priming signal. It should be emphasized, however, that gut-derived endotoxin is not the only source of Signal 1: even without measurable bacterial translocation, endogenous danger signals such as saturated fatty acids, ceramides, and cytokines released by lipotoxically stressed hepatocytes can prime the inflammasome by driving NF-κB–dependent transcription of NLRP3 and pro-IL-1β, which helps explain why inflammasome components are frequently up-regulated in MASLD livers even in the absence of overt endotoxemia (72).

#### 2.3.2 Signal 2 (activation signal)

The second signal, i.e., the activation or assembly signal, is triggered by a diverse array of seemingly unrelated danger factors, including extracellular ATP, uric acid crystals, cholesterol crystals, reactive oxygen species, and lysosomal rupture (41). Despite the distinct physicochemical nature of these stimuli, most converge on several common upstream events, such as potassium efflux, mitochondrial dysfunction, and reactive oxygen species accumulation (42, 43), which in turn promote NLRP3 oligomerization, the recruitment of ASC to form speck-like structures, and the subsequent recruitment of pro-caspase-1 to assemble a functional complex (44). Among these, NEK7 has been identified as an essential factor downstream of potassium efflux that mediates NLRP3 activation (45). This “diverse stimuli, common node” property renders NLRP3 a universal sensor for metabolic danger signals.

### 2.4 Downstream effector mechanisms of the NLRP3 inflammasome

#### 2.4.1 Maturation and secretion of IL-1β and IL-18

Activated caspase-1 cleaves the inactive precursors pro-IL-1β and pro-IL-18 into their mature forms and promotes their secretion. IL-1β is a potent pro-inflammatory mediator that recruits and activates neutrophils and monocytes/macrophages, amplifies local inflammation, and induces a variety of secondary inflammatory cytokines. As a core member of the IL-1 family, IL-1β exerts potent pro-inflammatory effects in both sterile and infectious inflammation (46); IL-18, in turn, functions in modulating adaptive immunity and amplifying tissue inflammation, and also participates in regulating energy metabolism—IL-18-deficient mice can develop hyperphagia, obesity, and insulin resistance, suggesting the complex, multifaceted roles of this cytokine in metabolism (47). Together, they constitute the principal downstream inflammatory output of NLRP3 and are the effector molecules that most targeting strategies ultimately aim to block.

#### 2.4.2 Pyroptosis

In addition to promoting cytokine maturation, activated caspase-1 also cleaves gasdermin D (GSDMD). Studies in 2015 identified GSDMD as a key substrate of inflammatory caspases; the N-terminal fragment released upon cleavage can oligomerize and form pores in the plasma membrane, leading to cell swelling, rupture, and the release of intracellular pro-inflammatory contents. This programmed necrotic process is termed pyroptosis (48, 49). As a lytic form of cell death, pyroptosis occupies an important position in the spectrum of hepatocyte death in metabolic liver disease (50). Pyroptosis is particularly consequential in this setting because, unlike apoptosis, it couples hepatocyte death directly to inflammation: a lipotoxically injured hepatocyte that might otherwise be cleared silently instead ruptures and discharges its contents, so each death event simultaneously eliminates a functional hepatocyte and recruits and activates neighboring immune and stellate cells; pyroptosis thus acts as a rate-limiting bridge between metabolic injury and progressive necroinflammation, and NLRP3-driven hepatocyte pyroptosis has been shown to promote hepatic inflammation and steatosis directly (202). In addition to pyroptosis, ferroptosis—iron-dependent lipid peroxidation-driven cell death—has also been demonstrated to be an important form of hepatocyte death that triggers inflammation in early NASH, forming a cell death network together with pyroptosis and other modes (51). GSDMD was subsequently confirmed as the executioner molecule of pyroptosis, with its N-terminal fragment being sufficient to independently induce pyroptosis and also required for the non-canonical release of IL-1β (52, 53). The cleaved GSDMD N-terminal fragment then binds to phospholipids on the plasma membrane, oligomerizes, and forms transmembrane pores, constituting the structural basis for membrane perforation and cell lysis in pyroptosis (54–56). Beyond the canonical pathway, cytosolic lipopolysaccharide from Gram-negative bacteria can directly activate caspase-11 (caspase-4/5 in humans) through the non-canonical pathway, which then cleaves GSDMD and indirectly promotes NLRP3 activation (57). In the terminal step of pyroptosis, NINJ1 has been shown to mediate the final rupture of the plasma membrane, thereby completing the release of cellular contents (58). Apart from GSDMD, other members of the gasdermin family (e.g., GSDME) can also execute pyroptosis and play roles in antitumor immunity (59). Unlike the “silent” clearance of apoptosis, pyroptosis is inherently highly pro-inflammatory; the released IL-1β, IL-18, ATP, HMGB1, and other molecules can further activate neighboring cells, forming a positive feedback loop that amplifies inflammation (50, 202).

### 2.5 Regulatory mechanisms of the NLRP3 inflammasome

To prevent excessive inflammation, NLRP3 activity is subject to multi-layered and fine-tuned regulation. At the post-translational modification level, phosphorylation, ubiquitination, and deubiquitination collectively determine the stability and assembly capacity of NLRP3 (60–63): NLRP3 phosphorylation is a requisite priming event for inflammasome activation (64), phosphorylation of the adaptor ASC serves as a molecular switch controlling speck-like aggregate formation (65), deubiquitination mediated by the deubiquitinase BRCC3 is a key prerequisite for its assembly (66), and the E3 ubiquitin ligase TRIM31 exerts negative regulation by promoting proteasomal degradation of NLRP3 (67). At the metabolic regulation level, the status of glucose and lipid metabolism can influence the activation threshold of NLRP3, indicating that its activity is tightly coupled to the cellular energy and nutrient state (44). Mitochondria serve as a central hub in NLRP3 regulation: reactive oxygen species and oxidized mitochondrial DNA released from damaged mitochondria are important activation signals; the externalization of cardiolipin, a phospholipid unique to the inner mitochondrial membrane, can directly bind to NLRP3 to promote its activation—collectively, damaged mitochondria release ROS, oxidized mtDNA, and externalized cardiolipin, all of which can promote NLRP3 activation (43, 68, 69). Meanwhile, thioredoxin-interacting protein (TXNIP) dissociates from thioredoxin under oxidative stress and instead binds to NLRP3, converting cellular redox imbalance into an inflammasome activation signal (70). These regulatory mechanisms are often disrupted in the metabolic stress environment of MASLD, constituting the molecular basis for sustained NLRP3 activation.

## 3 Activation mechanisms of the NLRP3 inflammasome in MASLD

**Figure 2:**
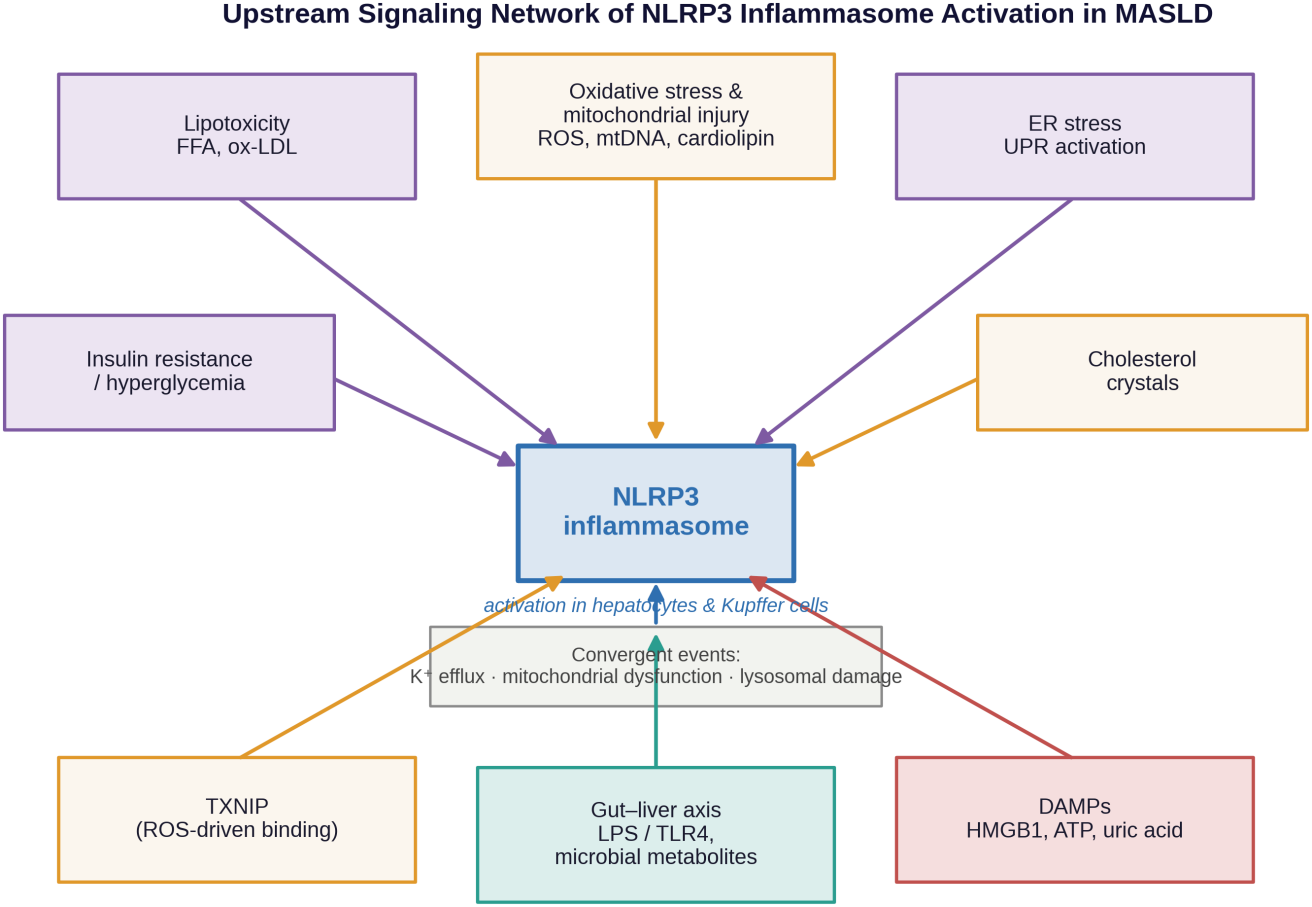
Upstream signaling network of NLRP3 inflammasome activation in MASLD. Convergence of lipotoxic (free fatty acids, ox-LDL), mitochondrial/oxidative (ROS, oxidized mtDNA, cardiolipin), endoplasmic-reticulum-stress, cholesterol-crystal, DAMP (HMGB1, ATP, uric acid), TXNIP, and gut–liver-axis (LPS, microbial metabolites) signals onto common activating nodes (K^+^ efflux, mitochondrial dysfunction, lysosomal damage) that trigger NLRP3 in hepatocytes and Kupffer cells. Arrows denote activating inputs; the signals shown coexist and reinforce one another *in vivo*.

In MASLD, the liver is situated in a complex stress environment shaped by lipid overload, oxidative imbalance, and gut-derived stimuli. The following sections delineate the major drivers of NLRP3 activation according to their signal origin. It should be emphasized that these signals often coexist and mutually reinforce one another *in vivo* rather than acting independently (**Figure 2**; **Table 1**).

**Table 1:** Activators, sources and mechanisms of NLRP3 inflammasome activation in MASLD.

Activator	Main source	Mechanism of NLRP3 activation	Pathological relevance in MASLD	References
Free fatty acids (FFA)	Lipolysis/diet	Mitochondrial ROS, ER stress	Core mediator of lipotoxicity-driven activation	(71–73)
Oxidized LDL (ox-LDL)	Lipid peroxidation	DAMP-mediated activation	Promotes hepatic inflammation and foam-cell formation	(74–76)
Reactive oxygen species	Mitochondria/NADPH oxidase	Triggers NLRP3 oligomerization	Bridge between oxidative stress and inflammation	(43, 68, 69)
Cardiolipin	Damaged mitochondria	Direct binding to NLRP3	Inflammatory signal of mitochondrial injury	(43, 68, 69)
ER stress	Endoplasmic reticulum	UPR-driven NLRP3 up-regulation	Lipotoxicity-induced cellular stress response	(82)
Cholesterol crystals	Lipid dysregulation	Lysosomal damage, DAMP signal	Marker/driver of MASH progression	(85–87)
HMGB1	Injured/necrotic hepatocytes	TLR4/NLRP3 activation	Amplifies inflammation after liver injury	(88, 89)
Extracellular ATP	Injured/pyroptotic cells	P2X7R–mediated K^+^ efflux	Amplifies post-pyroptosis inflammation	(90)
LPS	Gut microbiota (portal)	TLR4/NF-κB priming (signal 1)	Gut–liver axis inflammatory relay	(72, 95)
TXNIP	Hyperglycemia/lipid excess	Binds and activates NLRP3	Links redox imbalance to NLRP3	(70)

### 3.1 Lipotoxic signals

#### 3.1.1 Free fatty acid-induced NLRP3 activation

Excessive accumulation of free fatty acids lies at the core of lipotoxicity in NAFLD. Saturated fatty acids (e.g., palmitic acid) can promote NLRP3 activation through multiple pathways (71, 72): they enhance mitochondrial reactive oxygen species production, induce endoplasmic reticulum stress, and can be sensed as danger signals by immune cells. It is worth distinguishing that saturated and unsaturated fatty acids exert differential effects on the inflammasome—the former generally exhibit pro-inflammatory properties, whereas the latter can attenuate inflammatory responses under certain conditions (73). This difference partly explains the impact of dietary fat composition on disease progression.

#### 3.1.2 Oxidized low-density lipoprotein

Under conditions of exacerbated lipid peroxidation, oxidized low-density lipoprotein (ox-LDL) levels increase. Ox-LDL can be taken up by macrophages as a damage-associated molecular pattern, promoting foam cell formation while simultaneously activating the NLRP3 inflammasome (74), thereby amplifying local hepatic inflammation. Oxidized phosphatidylcholines also serve as endogenous lipid signals that activate macrophage NLRP3 (75). Furthermore, a Western diet and oxidized LDL can drive epigenetic reprogramming and “trained immunity” in myeloid progenitors via NLRP3, leading to long-term amplification of inflammatory responses (76), thus linking lipid metabolic disturbances to immune activation.

### 3.2 Oxidative stress and mitochondrial damage

Mitochondria are both the primary site of fatty acid oxidation and a major source of NLRP3-activating signals. Under lipid overload, the burden of mitochondrial β-oxidation increases and electron leakage from the respiratory chain intensifies, leading to excessive production of reactive oxygen species (ROS). Damaged mitochondria exhibit increased outer membrane permeability, allowing oxidized mitochondrial DNA (mtDNA) to leak into the cytoplasm and be recognized by NLRP3 as an activation signal. Meanwhile, cardiolipin, which is normally restricted to the inner mitochondrial membrane, translocates to the outer membrane upon damage and can directly bind to NLRP3 to promote its oligomerization. Thus, through multiple molecular events involving ROS, mtDNA, and cardiolipin, mitochondrial damage constitutes a key upstream driver of sustained NLRP3 activation in NAFLD (43, 68, 69, 77); mitochondrial ROS can also further amplify NLRP3 activation by inducing lysosomal damage (78). The clearance of damaged mitochondria depends on mitophagy. During the progression from NAFLD to NASH, impaired mitophagy leads to the accumulation of damaged mitochondria, which in turn persistently triggers NLRP3 inflammasome activation in hepatocytes (79). Conversely, NF-κB can limit excessive NLRP3 activation by promoting the autophagic clearance of damaged mitochondria, constituting an important negative feedback mechanism (80). Notably, in addition to activating NLRP3, mtDNA can also drive pro-inflammatory activation of macrophages via the cGAS-STING pathway, thereby exacerbating inflammation and fibrosis in NAFLD (81).

### 3.3 Endoplasmic reticulum stress

The accumulation of lipids and misfolded proteins in hepatocytes can trigger the unfolded protein response (UPR). There exists a bidirectional crosstalk between sustained endoplasmic reticulum (ER) stress and the NLRP3 inflammasome: multiple branches of the UPR can upregulate the expression of NLRP3 and pro-IL-1β, as well as promote inflammasome assembly by disturbing calcium homeostasis and aggravating oxidative stress. In NAFLD, ER stress, lipotoxicity, and oxidative stress converge, resulting in the continuous accumulation of inflammasome-activating signals (82).

### 3.4 Cholesterol crystals

Dysregulated cholesterol metabolism holds particular significance in the pathogenesis of NASH (83); indeed, NLRP3 activation by cholesterol crystals is also a core mechanism in atherosclerosis, suggesting that NASH and cardiovascular disease share this inflammatory pathway (84). When free cholesterol accumulates in hepatocytes beyond its solubility threshold, it can precipitate and form cholesterol crystals. These crystals, upon being phagocytosed by macrophages, cause lysosomal damage and subsequently activate NLRP3 as a damage-associated molecular pattern (74, 85). Cathepsin B, released upon lysosomal rupture, is a key mediator linking lysosomal damage to NLRP3 activation (86). Cholesterol crystal deposition is observed in NASH livers, and experimental studies suggest that NLRP3 activation triggered by cholesterol crystals in myeloid cells is a critical driver of hepatic inflammation. The NLRP3 inhibitor MCC950 can block this process and attenuate liver injury and fibrosis (87).

### 3.5 Damage-associated molecular patterns

As hepatocyte injury and pyroptosis occur, multiple damage-associated molecular patterns (DAMPs) are released, further intensifying inflammation. High mobility group box 1 (HMGB1) is released from injured or necrotic hepatocytes and acts as a classical DAMP that triggers sterile inflammation (88), providing inflammatory signals through receptors such as TLR4. Inflammasome activation and the active release of HMGB1 are also reciprocally coupled (89). Extracellular ATP induces potassium efflux via the P2X7 receptor to activate NLRP3 (90). Uric acid, which is elevated during purine metabolism dysregulation, triggers the inflammasome in its crystalline form (41). These DAMPs are largely produced by already damaged cells, thus forming a vicious cycle of “injury–inflammation–further injury” during advanced stages of the disease (72).

### 3.6 The gut microbiota–liver axis

#### 3.6.1 Gut microbiota dysbiosis in MASLD

The liver directly receives blood flow from the gut via the portal vein, resulting in its continuous exposure to gut-derived metabolites. NAFLD patients often exhibit alterations in gut microbiota composition and metabolic profiles (91, 92). This dysbiosis affects not only nutrient absorption and energy metabolism but also the nature and quantity of microbial-associated signals reaching the liver. Inflammasome functional deficiency can accelerate the progression of NAFLD and obesity through shaping gut dysbiosis, suggesting a role for microbiota–inflammasome interactions in disease evolution (93); among inflammasomes, NLRP6 plays a specific role in maintaining colonic microbial ecology and barrier integrity (94).

#### 3.6.2 Increased intestinal permeability and lipopolysaccharide translocation

Gut dysbiosis is frequently accompanied by impaired intestinal epithelial barrier function, a condition termed “leaky gut”. Barrier disruption facilitates the entry of lipopolysaccharide (LPS), a cell wall component of Gram-negative bacteria, into the liver via the portal vein, where it activates the TLR4–NF-κB pathway in Kupffer cells and hepatocytes. Gut-derived microbial nucleic acids can also promote steatohepatitis by inducing IL-1β production via TLR9 (95). This process precisely provides the indispensable priming signal (Signal 1) for the NLRP3 inflammasome, rendering the liver more susceptible to full inflammasome activation in the presence of a second signal such as lipotoxicity, thereby constituting an important mechanism through which the gut–liver axis promotes NAFLD progression.

#### 3.6.3 Regulation of NLRP3 by gut microbiota metabolites

The gut microbiota does not unidirectionally promote inflammation; its metabolites exert bidirectional modulation of NLRP3. Short-chain fatty acids, produced through the fermentation of dietary fiber, can regulate the inflammasome and promote intestinal homeostasis via metabolite-sensing receptors such as GPR43 and GPR109A (96), and in most circumstances help maintain intestinal barrier integrity and suppress excessive inflammation. Certain tryptophan metabolites can attenuate inflammasome activation through pathways such as the aryl hydrocarbon receptor. Notably, NAFLD patients have been shown to exhibit increased intestinal permeability and altered tight junction proteins, further exacerbating endotoxemia and intrahepatic inflammation (97). Bile acids regulate lipid and glucose metabolism as well as inflammation through receptors such as FXR and TGR5, and are important therapeutic targets in NAFLD (98). Bile acids also serve as key messengers in the gut–liver axis and can inhibit NLRP3 inflammasome activation via the farnesoid X receptor (FXR); conversely, disrupted bile acid metabolism can promote liver fibrosis through the NLRP3 pathway (99). Of note, different bile acids differentially activate NLRP3 in distinct hepatic cell types—lithocholic acid primarily activates Kupffer cells to trigger inflammation, whereas deoxycholic acid activates hepatic stellate cells to promote fibrosis, displaying marked cell-type specificity (100); chenodeoxycholic acid has also been demonstrated to activate the NLRP3 inflammasome and participate in cholestatic liver fibrosis (101). Alterations in gut microbiota composition simultaneously affect the production of these protective metabolites, thereby indirectly modulating the hepatic inflammatory state.

#### 3.6.4 Crosstalk between the gut microbiota and the NLRP3 inflammasome

Taken together, a bidirectional regulatory loop is formed between the gut microbiota and the NLRP3 inflammasome: the microbiota and its metabolites influence NLRP3 activation, while NLRP3-mediated intestinal inflammation in turn shapes microbial community structure and barrier function (93, 94). This loop provides a potential therapeutic entry point for modulating NLRP3 activation through manipulation of the gut microbiota.

### 3.7 The TXNIP-mediated activation pathway

TXNIP is a representative molecule linking oxidative stress to NLRP3 activation. Under physiological conditions, TXNIP binds to thioredoxin and remains inactive; when ROS levels rise, the two dissociate, and free TXNIP instead binds to NLRP3 to promote its activation (70). High-glucose and high-lipid environments can induce TXNIP upregulation, and TXNIP-deficient mice exhibit attenuated inflammasome activation accompanied by improved glucose tolerance and insulin sensitivity, suggesting that the TXNIP–NLRP3 axis is a key node connecting metabolic dysregulation to inflammation. This axis is particularly important in the context of NAFLD with concomitant insulin resistance.

## 4 Role of the NLRP3 inflammasome in MASLD progression

### 4.1 Transition from simple steatosis to MASH

#### 4.1.1 NLRP3 as a key pro-inflammatory mediator

In the simple steatosis stage, marked lipid accumulation is present in hepatocytes, yet inflammation remains inconspicuous, and this stage is reversible with appropriate intervention. Activation of the NLRP3 inflammasome is considered a critical event that pushes the disease beyond this reversible threshold toward NASH (102). Genetic evidence strongly supports this notion: in mice, induced gain-of-function activation of NLRP3 leads to early-onset and severe diet-induced NASH, whereas NLRP3 knockout protects mice from the inflammatory and fibrotic injury of NASH (103, 104). Excessive activation of the NLRP3 inflammasome is also associated with obesity-related systemic inflammation and insulin resistance, suggesting a common mechanistic basis in metabolic and hepatic pathologies (105, 106). Mice deficient in inflammasome components (NLRP3, ASC, or caspase-1) are resistant to high-fat diet-induced obesity, hepatic steatosis, and insulin resistance, further supporting a causal role of this pathway in metabolic disorders (107). Mice carrying activating mutations in NLRP3 develop spontaneous liver fibrosis even on a normal diet, further demonstrating that excessive NLRP3 activation alone is sufficient to drive the hepatic inflammation–fibrosis process.

#### 4.1.2 The IL-1β/IL-18-driven inflammatory microenvironment

**Figure 3:**
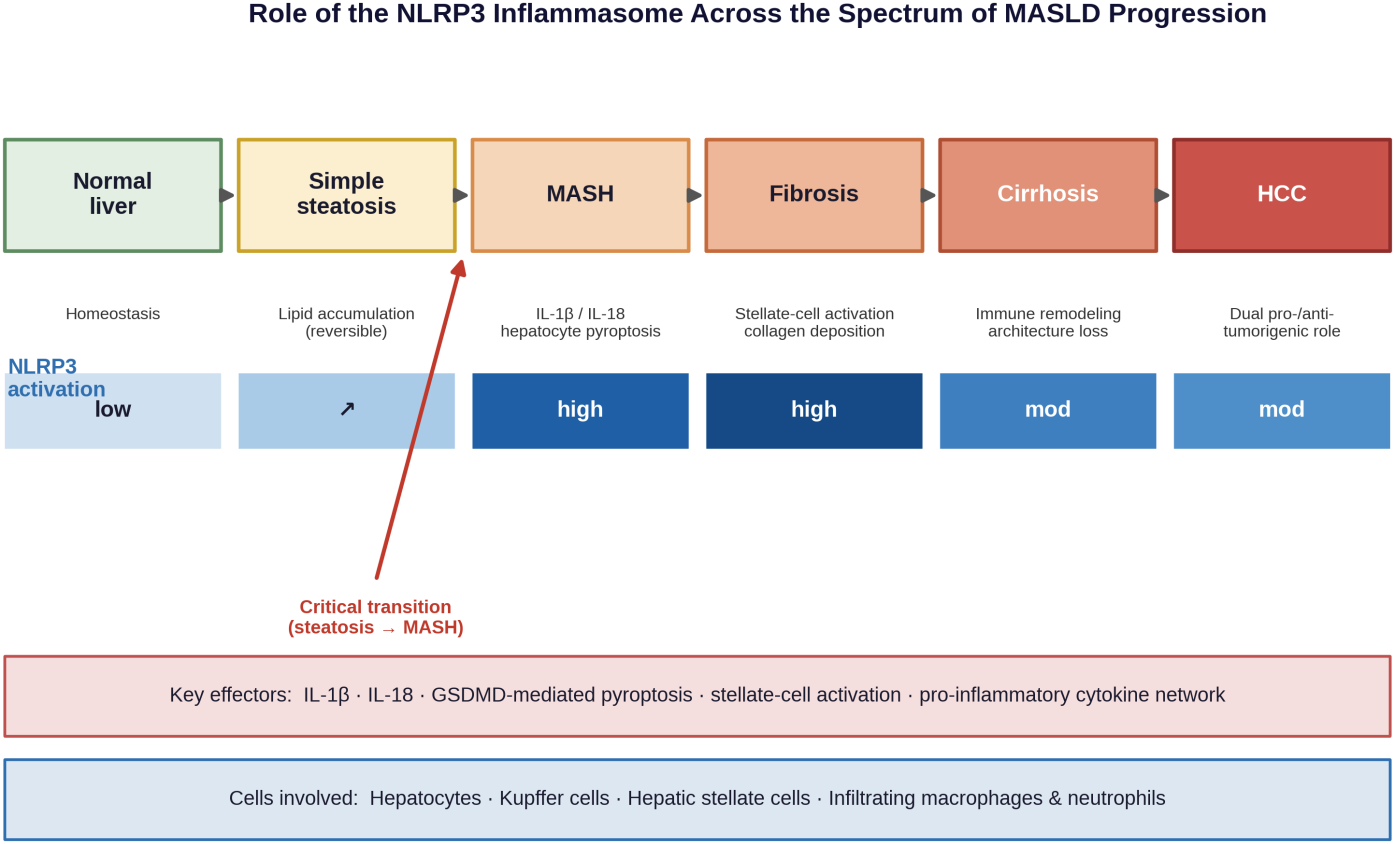
Role of the NLRP3 inflammasome across the spectrum of MASLD progression. Stage-wise contribution of NLRP3 activation from simple steatosis → steatohepatitis (MASH) → fibrosis → cirrhosis → hepatocellular carcinoma, indicating the dominant NLRP3-associated cell types and effector outputs (IL-1β/IL-18 release, hepatocyte pyroptosis, stellate-cell activation, immune remodeling) at each step and the context-dependent, dual pro-/anti-tumorigenic role at the HCC stage.

The IL-1β and IL-18 released upon NLRP3 activation shape an inflammatory microenvironment conducive to disease progression (**Figure 3**). These cytokines recruit neutrophils and monocytes/macrophages to infiltrate the liver and induce the production of secondary inflammatory mediators such as TNF-α and IL-6, forming a mutually reinforcing pro-inflammatory network. The sustained infiltration and activation of immune cells not only directly injure hepatocytes but also provide the signaling basis for subsequent fibrotic responses—multiple intrahepatic intercellular communication circuits tightly couple metabolic injury to inflammation and fibrosis (108). At the innate immune level, liver macrophages comprise self-maintaining Kupffer cells and monocyte-derived macrophages, with their polarization state dictating the trajectory of inflammation (109). Single-cell sequencing has revealed the emergence of a “NASH-associated macrophage” (NAM) subset, marked by high TREM2 expression, in NASH livers, with its abundance correlating with disease severity (110); these lipid-associated macrophages regulate metabolic homeostasis in a TREM2-dependent manner and express osteopontin (111, 112). Single-cell and spatial proteogenomics have further resolved the cellular niches that drive fibrosis in human cirrhosis (113), delineating the dynamic changes in the composition of resident and recruited macrophages (114), the impact of impaired Kupffer cell self-renewal on responses to lipid overload (115), niche-specific epigenetic reprogramming (116), and evolutionarily conserved hepatic macrophage niches (117). In NAFLD, liver sinusoidal endothelial cells undergo defenestration (capillarization) and shift to a pro-inflammatory phenotype, participating in leukocyte recruitment through the upregulation of adhesion molecules and chemokines, thereby amplifying intrahepatic inflammation (118, 119). At the adaptive immune level, intrahepatic autoreactive CXCR6^+^CD8 T cells can sense extracellular ATP via the P2X7 receptor and exert antigen-nonspecific killing of hepatocytes (120); gut-derived B cells and regulatory T cells also contribute to NASH-associated inflammation and fibrosis through IgA-FcR signaling and amphiregulin, respectively (121, 122). Moreover, even without undergoing cell death, hepatocytes under lipotoxic stress can release extracellular vesicles carrying pro-inflammatory signals to recruit and activate macrophages, rendering sublethal hepatocyte injury an important driver of hepatic inflammation (123, 124). The ubiquitination network also participates in this inflammatory regulation; for example, the deubiquitinase A20 (TNFAIP3) can alleviate NASH by inactivating hepatic ASK1 (125).

#### 4.1.3 Clinical relevance

Clinical observations corroborate the above mechanisms. The expression of NLRP3 and its downstream molecules is elevated in the liver tissue and peripheral blood of NAFLD/NASH patients, and the expression of genes related to NLRP3, ASC, and caspase-1 is significantly increased in NASH patients. The activated form of caspase-1 can be detected in the serum of NAFLD patients, and its level correlates with disease severity. In patients with severe NAFLD, elevated expression of NLRP3 and IL-1β correlates with the liver fibrosis marker COL1A1, suggesting an association between the degree of inflammasome activation and the severity of steatosis and fibrosis (104).

### 4.2 Hepatocyte pyroptosis and liver injury

#### 4.2.1 Pathological significance of pyroptosis

Pyroptosis is a pro-inflammatory mode of cell death distinct from apoptosis. Apoptosis is typically characterized by an intact plasma membrane and the absence of cellular content leakage, representing a form of “silent” death; in contrast, pyroptosis involves pore formation in the plasma membrane, cell swelling and rupture, and the massive release of intracellular pro-inflammatory contents. In NAFLD, hepatocyte pyroptosis not only directly causes hepatic parenchymal injury and elevated transaminase levels, but also ignites surrounding inflammatory responses through the release of DAMPs, resulting in a continuously expanding zone of injury.

#### 4.2.2 Key molecular mechanisms of pyroptosis

**Figure 4:**
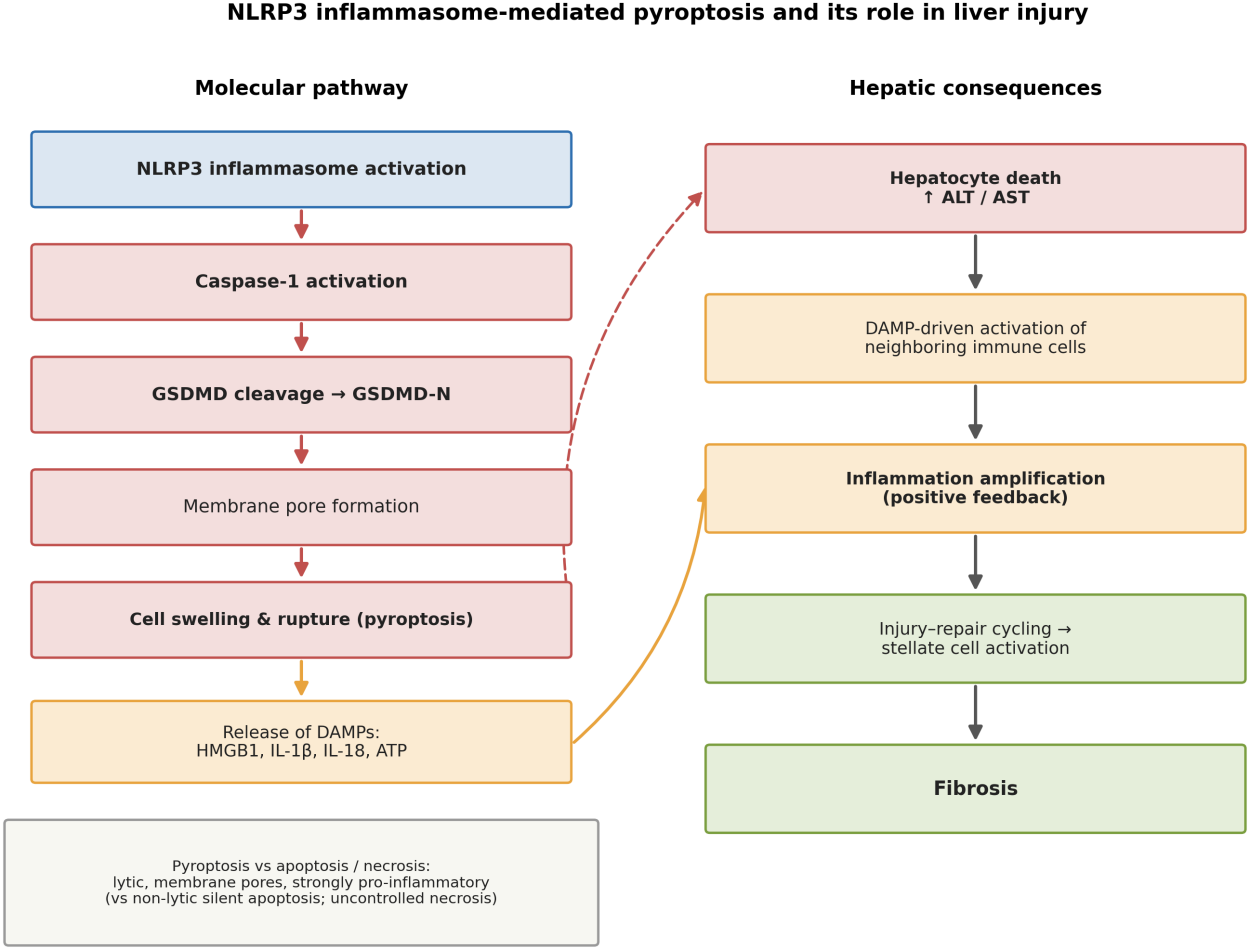
NLRP3 inflammasome-mediated pyroptosis pathway and its role in liver injury. The NLRP3–caspase-1–GSDMD axis in hepatocytes: active caspase-1 cleaves GSDMD, and the GSDMD-N fragment oligomerizes to form plasma-membrane pores, releasing IL-1β, IL-18, and DAMPs (HMGB1, ATP). The released DAMPs activate neighboring immune cells and amplify inflammation through a positive-feedback loop, and the resulting injury–repair cycling drives hepatic stellate-cell activation and fibrosis. The lytic, strongly pro-inflammatory nature of pyroptosis is contrasted with non-lytic silent apoptosis and uncontrolled necrosis.

The core of hepatocyte pyroptosis is the NLRP3 inflammasome–caspase-1–GSDMD axis. Upon NLRP3 activation, active caspase-1 cleaves GSDMD, and the resulting N-terminal fragment forms pores in the plasma membrane, ultimately leading to pyroptosis (48, 52). The central role of GSDMD in pyroptosis execution has been validated in liver disease: the N-terminal fragment of GSDMD is markedly upregulated in the livers of NASH patients; GSDMD deficiency attenuates the steatohepatitis phenotype in mice, including improvements in inflammation, steatosis, and fibrosis, whereas GSDMD overexpression exacerbates hepatic inflammation and lipid accumulation (126) (**Figure 4**).

#### 4.2.3 Association between pyroptosis and NAFLD severity

The expression of pyroptosis-related molecules parallels the histological severity of NAFLD. As the disease advances from simple steatosis to steatohepatitis and fibrosis, the levels of GSDMD and its downstream inflammatory factors increase correspondingly, indicating that inflammatory cell death, represented by pyroptosis, is an important marker and driver of disease progression.

### 4.3 Liver fibrosis

#### 4.3.1 The central role of NLRP3 in fibrosis

Liver fibrosis is the core determinant of adverse outcomes in NAFLD (127): multiple long-term follow-up cohorts and meta-analyses consistently demonstrate that fibrosis stage—rather than steatosis alone or NAFLD activity score—is the strongest predictor of all-cause and liver-related mortality (128–132), and activation of the NLRP3 inflammasome plays an indispensable role in the fibrotic process. In NLRP3 knockout mice, the development of fibrosis is markedly suppressed; the conclusion that NLRP3 activation is required for the development of NAFLD-related fibrosis has been validated by genetic approaches in multiple models (104). The major pathways through which NLRP3 promotes fibrosis include the following.

#### 4.3.2 Mechanism 1: activation of hepatic stellate cells by pro-inflammatory cytokines

Quiescent hepatic stellate cells (HSCs) are the key effector cells in liver fibrosis, transdifferentiating from a quiescent phenotype into contractile, collagen-producing myofibroblasts in response to injury signals (133). Danger signals of hepatocyte mitochondrial origin (e.g., mtDNA) can directly act on HSCs and drive fibrosis progression (134). IL-1β, a downstream product of NLRP3, and TNF-α generated through amplified inflammation can activate HSCs, promoting their proliferation and transdifferentiation into myofibroblasts, which in turn synthesize large quantities of collagen and other extracellular matrix components. This inflammatory mediator-driven HSC activation is the direct source of fibrotic deposition.

#### 4.3.3 Mechanism 2: sustained inflammation and the injury–repair cycle

NLRP3-mediated hepatocyte pyroptosis and sustained inflammation constitute a repetitive injury–repair cycle. Each round of hepatocyte death is accompanied by DAMP release and inflammatory cell recruitment, while the matrix deposited during the repair process is difficult to fully clear. As the cycle repeats, fibrotic tissue accumulates progressively, ultimately leading to irreversible alterations in hepatic architecture. Notably, inflammasome particles released from pyroptotic hepatocytes can be taken up by HSCs and directly promote their activation, providing a direct molecular link between pyroptosis and fibrosis (135).

#### 4.3.4 Mechanism 3: NLRP3 activation in HSCs themselves

In addition to the paracrine effects of inflammatory mediators, HSCs themselves express a functional NLRP3 inflammasome. Endogenous NLRP3 activation in HSCs can directly promote their activation and fibrotic phenotype (136), implying that NLRP3 not only drives fibrosis indirectly via immune cells but also functions within the fibrotic effector cells themselves. Based on this, targeting NLRP3 or NLRP3-mediated pyroptosis in HSCs offers a cell-specific potential strategy for antifibrotic therapy.

### 4.4 Hepatocellular carcinoma

#### 4.4.1 Association between chronic inflammation and liver cancer

Against the background of NASH-related chronic inflammation, the liver can progressively advance to hepatocellular carcinoma (HCC) via cirrhosis; in recent years, NASH has become one of the fastest-growing etiologies of HCC worldwide (137, 138). A sustained inflammatory microenvironment provides the soil for the malignant transformation of hepatocytes, and NLRP3-mediated inflammation is considered to participate in this process.

#### 4.4.2 Possible mechanisms of NLRP3 in hepatocarcinogenesis

The possible mechanisms by which NLRP3 contributes to hepatocarcinogenesis include: persistent inflammation and oxidative stress can cause DNA damage and genomic instability, thereby increasing the risk of malignant transformation; inflammatory cytokines can aberrantly activate signaling pathways related to cell proliferation and survival; and inflammasome activation may also participate in shaping an immunosuppressive tumor microenvironment, weakening antitumor immune surveillance. These mechanisms often interweave, collectively driving the evolution from chronic inflammation to neoplasia.

#### 4.4.3 The dual role of NLRP3 in liver cancer

Caution is warranted, however, as the role of NLRP3 in liver cancer is markedly context-dependent. On the one hand, neutrophil extracellular traps (NETs) form in NASH livers and create a pro-tumorigenic microenvironment, contributing to the progression from NASH to HCC (139). At the adaptive immune level, NAFLD can lead to the selective loss of CD4^+^ T lymphocytes, thereby promoting hepatocarcinogenesis (140); functionally exhausted CD8^+^PD1^+^ T cells also accumulate in NASH livers, impairing antitumor immune surveillance and reducing the efficacy of immune checkpoint therapy (141). Moreover, platelets accumulate in NASH livers via GPIbα and promote immune cell recruitment, and antiplatelet therapy can alleviate NASH and suppress subsequent hepatocarcinogenesis (142); inflammation-induced IgA^+^ plasma cells can subvert CD8^+^ T cell immunity against liver cancer (143); endoplasmic reticulum stress and overnutrition can also cooperate to trigger spontaneous HCC through a TNF-dependent pathway (144). The complex relationship between NAFLD/NASH and liver cancer, characterized by both inflammatory drive and immunosuppression, as well as the overall landscape of the liver cancer immune microenvironment, have been systematically reviewed (145, 146). In a setting of chronic inflammation, NLRP3 activation can promote tumor initiation and progression; on the other hand, in established tumors, pyroptosis—as a form of immunogenic cell death—may enhance antitumor immunity and exert a tumor-suppressive effect. For instance, estrogen can upregulate the NLRP3 inflammasome via ERβ and trigger pyroptosis in HCC cells, thereby inhibiting tumor growth, suggesting that NLRP3 can have a tumor-suppressive effect under specific circumstances (147). This dual pro-tumorigenic and anti-tumorigenic nature implies that interventions targeting NLRP3 require differential considerations at different stages of liver cancer, as simple inhibition or activation may each produce opposite consequences.

## 5 Therapeutic strategies targeting the NLRP3 inflammasome

### 5.1 Theoretical basis for NLRP3 as a therapeutic target

**Figure 5:**
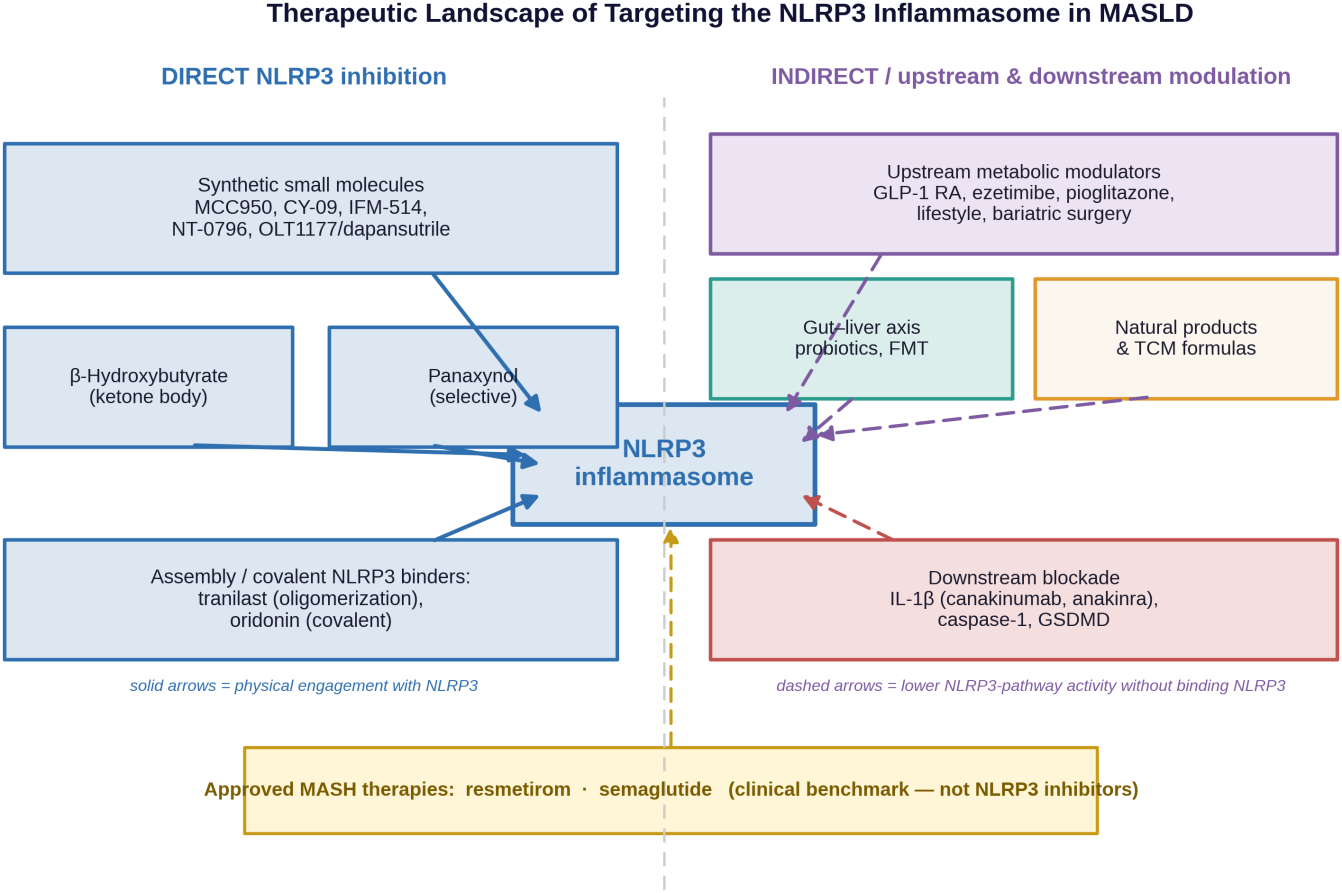
Therapeutic landscape of targeting the NLRP3 inflammasome in MASLD. Intervention points are classified by their mechanism of engagement with NLRP3. Direct NLRP3 inhibitors physically engage NLRP3 or its immediate assembly and comprise synthetic small molecules (MCC950, CY-09, IFM-514, NT-0796, OLT1177/dapansutrile) together with the ketone body β-hydroxybutyrate and the selective natural product panaxynol. Indirect modulators lower NLRP3-pathway activity without binding NLRP3 and comprise upstream metabolic modulators (including the approved MASH therapies resmetirom and semaglutide), gut–liver-axis interventions, most natural products and traditional-medicine formulas, and downstream IL-1β/caspase-1/GSDMD blockade. The approved MASH agents are included for clinical context and are not themselves NLRP3 inhibitors.

The NLRP3 inflammasome has become an attractive therapeutic target because of its pivotal position in the pathological network of NAFLD. As multiple metabolic danger signals ultimately converge on NLRP3, inhibition of this node is expected to simultaneously curb downstream inflammatory amplification and fibrotic progression, without the need to individually target each upstream stimulus (148, 149). Furthermore, because NLRP3 inhibition acts on the host’s own inflammatory pathways rather than on pathogens, it does not raise the issue of pathogen resistance. That said, as an integral component of innate immunity, complete or long-term inhibition of NLRP3 may also raise concerns such as increased susceptibility to infection; therefore, how to strike a balance between efficacy and safety is a core issue in drug development in this field To make this evidence base easier to weigh, the strategies below are grouped by molecular target and, within each group, ordered by level of evidence, while a consistent distinction is drawn between direct and indirect evidence (**Table 2**). Direct evidence comes from agents that physically engage NLRP3 or its immediate assembly (for example MCC950, CY-09, and other selective inhibitors), whereas indirect evidence comes from metabolic, antioxidant, gut-directed, or anti-inflammatory interventions that lower NLRP3-pathway markers without binding NLRP3 itself; the two should not be equated. On this basis the sections below cover, in turn, direct small-molecule NLRP3 inhibitors (5.2), natural-product and traditional-medicine modulators of the pathway (5.3 and 5.4), upstream metabolic modulators together with the therapies now clinically approved for MASH (5.5.1), gut–liver-axis interventions (5.5.2), and downstream cytokine, caspase, and GSDMD blockade (5.5.3), with the clinical stage and translational relevance of each noted where the evidence allows (**Figure 5**).

**Table 2:** Representative drugs/compounds targeting the NLRP3 inflammasome and their development stages.

Compound/drug	Class	Target/mechanism	Effect in MASLD/MASH model	Stage	Ref.
MCC950	Synthetic small molecule	Binds NLRP3 NACHT domain (direct)	↓ hepatic inflammation, ↓ fibrosis	Preclinical	(87, 150)
IFM-514	Synthetic small molecule	Specific NLRP3 antagonist (direct)	↓ NASH inflammation & fibrosis	Preclinical	(156)
NT-0796	Synthetic small molecule	Brain-penetrant NLRP3 inhibitor (direct)	Improves cardiometabolic risk factors	Phase I/II	(157)
20(S)-protopanaxatriol	Natural product (triterpene)	Inhibits NLRP3 inflammasome (indirect)	Improves MAFLD (↓ inflammation/fibrosis)	Preclinical	(162)
Panaxynol	Natural product (Panax ginseng)	Selective NLRP3 inhibition (direct)	↓ NASH liver injury, ↓ pyroptosis	Preclinical	(163)
Paeoniflorin	Natural product (flavonoid/glycoside)	STING–NLRP3 axis (indirect)	↓ hepatocyte pyroptosis in MAFLD	Preclinical	(164)
Hyperoside	Natural product (flavonoid)	Flot2/TLR4/NLRP3 in HSC (indirect)	↓ NASH-associated fibrosis	Preclinical	(166)
Ginger essential oil	Natural product	NLRP3 + gut microbiota-LPS-TLR4 (indirect)	Prevents NASH progression	Preclinical	(170)
Sulforaphane	Natural product (isothiocyanate)	AMPK-autophagy → ↓ NLRP3 (indirect)	↓ HFD-induced steatosis	Preclinical	(171)
Rhubarb free anthraquinones	TCM active components	↓ ASC oligomerization/NLRP3 (indirect)	↓ hepatic inflammation & steatosis	Preclinical	(172)
Berberine	TCM alkaloid	ROS/TXNIP → ↓ NLRP3/pyroptosis (indirect)	↓ NASH inflammation	Preclinical	(173)
Diosgenin	Natural product (steroidal saponin)	Hepatic NLRP3-dependent pathway (indirect)	Improves MAFLD	Preclinical	(161)
Liraglutide	Metabolic modulator (GLP-1 RA)	Mitophagy → ↓ NLRP3/pyroptosis (indirect)	↓ hepatic inflammation	Approved for T2D/obesity	(176)
Ezetimibe	Metabolic modulator	AMPK-TFEB autophagy → ↓ NLRP3 (indirect)	↓ NASH	Approved (lipid-lowering)	(175)
Canakinumab	Biologic	Anti-IL-1β mAb (downstream blockade) (indirect)	Indirect NLRP3 downstream blockade	Approved (CV indication)	(188)

Engagement with NLRP3: “direct” denotes agents shown to bind NLRP3 or its immediate assembly (MCC950, IFM-514, NT-0796, and the selective natural product panaxynol); “indirect” denotes upstream metabolic, gut-directed, antioxidant, or downstream interventions that lower NLRP3-pathway markers without demonstrated NLRP3 engagement. The Stage column refers to each agent’s overall regulatory status, which may differ from its status specifically for MASLD/MASH; for the natural products and traditional-medicine constituents listed, efficacy in MASLD/MASH remains preclinical.

“↓” (downward arrow) indicates reduction, suppression, or inhibition. “→” (rightward arrow) indicates “leads to,” “results in,” or “causes” a subsequent event.

### 5.2 Synthetic inhibitors

#### 5.2.1 MCC950

MCC950 is the most extensively studied small-molecule NLRP3 inhibitor. Initially reported as a potent and specific NLRP3 inhibitor (150), it binds to the NACHT domain of NLRP3 and interferes with an ATP hydrolysis motif, thereby preventing NLRP3 activation (151). MCC950 has demonstrated clear efficacy in animal models of NASH: in two mouse models of steatohepatitis, MCC950 reduced hepatic caspase-1 and IL-1β levels, attenuated liver inflammation, and ameliorated fibrosis, an effect partly attributed to its blockade of NLRP3 activation triggered by cholesterol crystals in myeloid cells (87). Despite robust mechanistic evidence, the early clinical development of MCC950 was hampered by safety concerns, indicating that optimized molecular design is still required for translation from preclinical to clinical settings.

#### 5.2.2 Other small-molecule inhibitors

Following MCC950, a variety of NLRP3 inhibitors with distinct chemical structures and mechanisms of action have been successively developed, such as CY-09, which blocks ATP binding; tranilast, which interferes with NLRP3 oligomerization; and the covalent inhibitor oridonin (152–154). Among these, CY-09 has been shown to reduce hepatic steatosis in experimental NAFLD mice (155). IFM-514, a specific NLRP3 antagonist, reduced hepatic inflammation and fibrosis, lowered portal pressure, and was accompanied by marked suppression of caspase-1 activation in a NASH model established in ApoE-deficient mice (156). NT-0796 is an orally bioavailable, brain-penetrant, potent NLRP3 inhibitor (an ester prodrug that is converted to the active metabolite NDT-19795 by carboxylesterase-1) and has entered clinical development for inflammatory and neuroinflammatory diseases (157). In addition, the oral selective NLRP3 inhibitor OLT1177 (dapansutrile), a β-sulfonyl nitrile compound with a favorable safety profile in humans (158), has demonstrated good safety and preliminary efficacy in Phase 2a clinical trials of inflammatory diseases such as acute gout flares, lending support to the clinical translation of this drug class (159). It should be objectively noted that some studies have failed to reproduce the improvement of experimental NASH by pharmacological NLRP3 inhibition in certain models, suggesting that efficacy may depend on the model, dosing regimen, and exposure level, and that the definitive value of these candidate drugs in the NAFLD/NASH population remains to be confirmed by sufficient clinical evidence.

#### 5.2.3 Current status of clinical research

Overall, clinical research on small molecules directly targeting NLRP3 in the field of NAFLD remains in its infancy, with a limited number of registered trials conducted to date. In recent years, strategies combining NLRP3 inhibition with metabolic therapies that have shown benefit (e.g., GLP-1 receptor agonists) have attracted attention. The combination of NT-0796 with semaglutide exemplifies this approach: in a diet-induced obesity model, NT-0796 enhanced and sustained the weight-loss effect mediated by GLP-1 receptor agonists, providing preclinical evidence for combination regimens of metabolic therapy and NLRP3 inhibition (160); however, the definitive clinical value of such combination strategies still requires validation in prospective studies.

### 5.3 Natural product-derived modulators of the NLRP3 pathway

#### 5.3.1 Terpenoids and saponins

On current evidence, most of the natural products discussed below are best regarded as indirect modulators of the NLRP3 pathway: with few exceptions (for example the selective inhibitor panaxynol), they are shown to lower NLRP3, caspase-1, or IL-1β levels rather than to physically engage NLRP3. Diosgenin, a steroidal saponin, has been reported to ameliorate MAFLD through NLRP3 inflammasome-dependent signaling pathways in the liver, reducing the expression of NLRP3, caspase-1, and IL-1β in a high-fat diet model and thus being regarded as an indirect modulator of the NLRP3 pathway (161). Among the active constituents derived from ginseng, 20(S)-protopanaxatriol can inhibit NLRP3 inflammasome activation in multiple primary cell types, including macrophages, hepatocytes, and Kupffer cells, and improves liver function while attenuating hepatic inflammation and fibrosis in MCD diet-induced MAFLD mice (162); panaxynol has also been demonstrated to selectively inhibit the NLRP3 inflammasome, blocking IL-1β secretion and pyroptotic cell death, thereby alleviating NASH-associated liver injury (163).

#### 5.3.2 Flavonoids

Flavonoids have been extensively studied for their anti-inflammatory and hepatoprotective properties. Paeoniflorin has been reported to suppress NLRP3 inflammasome-mediated hepatocyte pyroptosis by targeting STING, thereby ameliorating MAFLD, with its effect dependent on the regulation of the STING–NLRP3 axis (164); this finding resonates with the mechanisms by which STING promotes hepatic inflammation and fibrosis in NAFLD (81, 165). Hyperoside has been demonstrated to directly bind to Flot2 and block the Flot2–TLR4 interaction in lipid rafts, thereby inhibiting TLR4/NLRP3-mediated pyroptosis in hepatic stellate cells, and to exert anti-NASH fibrotic effects by remodeling the gut microbiota and reducing LPS (166). Quercetin can also alleviate high-fat diet-induced NAFLD and suppress NLRP3-associated inflammation by correcting gut microbiota dysbiosis and the associated activation of the gut–liver axis (167); dihydromyricetin has been shown to mitigate NAFLD by inhibiting the NLRP3 inflammasome and ameliorating oxidative stress and lipid metabolic disturbances (168); apigenin attenuates hepatic lipid accumulation and inflammation in a model of atherosclerosis combined with NAFLD by inhibiting the NLRP3 inflammasome (169). Alpinetin, the principal flavonoid of Alpinia katsumadai, likewise ameliorates carbon tetrachloride–induced liver injury and fibrosis by activating the Nrf2 pathway and suppressing the NLRP3–ASC–caspase-1 axis, lowering hepatic NLRP3, cleaved caspase-1, IL-1β, and IL-18 (203); although this evidence derives from a toxin-induced fibrosis model rather than a dietary MASH model, it reinforces the anti-fibrotic potential of flavonoid-based NLRP3 inhibition.

#### 5.3.3 Phenolic compounds and others

Among phenolic and volatile constituents, the ginger active component 6-shogaol has been reported to alleviate hepatic inflammation and fibrosis in mice fed a methionine-choline-deficient diet by suppressing oxidative stress, endoplasmic reticulum stress, and multiple cell death pathways (170). Such multi-pronged effects—simultaneously inhibiting the inflammasome and modulating the gut microbiota—represent a potential advantage of natural products over single-target small molecules. Furthermore, sulforaphane, an isothiocyanate derived from cruciferous vegetables, can inhibit hepatic NLRP3 inflammasome activation via the AMPK–autophagy axis and attenuate high-fat diet-induced hepatic steatosis and inflammation (171).

#### 5.3.4 Limitations and prospects of natural product research

A common problem in current natural product research is that the majority of evidence originates from animal models and *in vitro* experiments; the exact structures, dose–response relationships, and bioavailability of the active constituents remain unclear, and clinical evidence is even more scarce. Future efforts need to proceed on the basis of well-defined active constituents and molecular targets, and require structural optimization and rigorous clinical evaluation to achieve the translation from experimental findings to clinical applications.

### 5.4 Traditional Chinese medicine interventions

#### 5.4.1 Active constituents of Chinese materia medica

As with the natural products above, the traditional-medicine constituents and formulas below act, on current evidence, as indirect pathway modulators: they reduce NLRP3, caspase-1, and IL-1β without demonstrated direct binding to NLRP3. At the level of single constituents, rhubarb free anthraquinones (including rhein, emodin, and aloe-emodin) have been shown to ameliorate NAFLD in mice by inhibiting ASC oligomerization and blocking NLRP3 inflammasome activation, thereby reducing serum transaminases, IL-1β, hepatic inflammation, and fibrosis (172). Berberine, an isoquinoline alkaloid, can inhibit NLRP3 inflammasome activation and hepatocyte pyroptosis via the ROS/TXNIP axis, thereby improving NASH (173); its derivative demethyleneberberine has also been demonstrated to alleviate NAFLD by inhibiting the NLRP3 inflammasome and oxidative stress (174). Such studies establish a link between the active substances of traditional Chinese medicines and well-defined inflammasome mechanisms, thereby facilitating the modernization of TCM research.

#### 5.4.2 Chinese herbal formulas

At the formula level, a variety of Chinese herbal formulas based on therapeutic principles such as clearing heat and draining dampness, activating blood and resolving stasis, or soothing the liver and strengthening the spleen have been used for NAFLD/NASH in both experimental and clinical settings, and are believed to exert anti-inflammatory effects partly through inhibiting the NF-κB/NLRP3 pathway. The multi-component nature of formulas enables them to simultaneously act on multiple aspects such as metabolism, inflammation, and the gut microbiota; however, their precise active constituents and molecular targets still require elucidation through high-quality studies.

#### 5.4.3 The multi-target advantage of TCM in regulating NLRP3

The hallmark of TCM interventions in NAFLD lies in their multi-component, multi-target, and holistic regulatory characteristics, which align with the “multiple parallel hits” pathogenesis of NAFLD. Nevertheless, the complexity of formula composition, the difficulty in fully elucidating their mechanisms of action, and the paucity of high-quality clinical evidence remain the major bottlenecks restricting their widespread acceptance.

### 5.5 Other intervention strategies

#### 5.5.1 Metabolic modulators

Improving the upstream metabolic state to indirectly suppress NLRP3 represents a class of strategies already grounded in clinical evidence. Ezetimibe, while lowering cholesterol, has been shown to inhibit the NLRP3 inflammasome through AMPK-TFEB-mediated autophagy activation, thereby ameliorating experimental steatohepatitis (175). The GLP-1 receptor agonist liraglutide can promote mitophagy to clear damaged mitochondria and reduce ROS production, consequently inhibiting NLRP3 inflammasome activation and hepatocyte pyroptosis and delaying NASH progression (176). These drugs are already in clinical use for metabolic diseases, and their modulation of NLRP3 provides a new mechanistic explanation for their hepatoprotective effects. At a broader level of metabolic intervention, robust randomized controlled trial evidence already exists: significant weight loss achieved through lifestyle intervention improves the histological features of NASH (177, 178); for patients with severe obesity, bariatric surgery can yield durable (5-year) NASH resolution and fibrosis regression (179), and is associated with a significantly reduced risk of adverse hepatic and cardiovascular outcomes (180); pioglitazone improves liver histology in NASH patients (181, 182); the GLP-1 receptor agonists liraglutide and semaglutide increased NASH resolution rates in the LEAN and Phase 2 NASH trials, respectively (183, 184); and the thyroid hormone receptor β agonist resmetirom, in the Phase 3 MAESTRO-NASH trial, significantly increased the rate of NASH resolution and improved fibrosis, becoming the first approved therapeutic agent for NASH (185). The clinical landscape has since moved further: resmetirom (Rezdiffra) received FDA approval in March 2024 for non-cirrhotic MASH with moderate-to-advanced (F2–F3) fibrosis, and in August 2025 the GLP-1 receptor agonist semaglutide (Wegovy 2.4 mg) became the second—and first GLP-1-based—agent approved for the same indication, on the strength of the Phase 3 ESSENCE trial, in which 62.9% of treated patients achieved resolution of steatohepatitis and 37.0% showed fibrosis improvement without worsening of steatohepatitis, versus 34.3% and 22.5% on placebo (204). Neither agent is a direct NLRP3 inhibitor; both are included here as essential current clinical context, because by relieving upstream lipotoxicity, adiposity, and insulin resistance they are expected to reduce the metabolic drive on NLRP3, and they now set the benchmark against which any NLRP3-directed therapy will have to be judged. All of these metabolic therapies can indirectly reduce the activation burden on NLRP3 by ameliorating upstream lipotoxicity and insulin resistance. Notably, the ketone body β-hydroxybutyrate, produced during caloric restriction and the ketogenic state, can directly block NLRP3 inflammasome activation, providing a molecular explanation for the anti-inflammatory benefits of lifestyle interventions (186).

#### 5.5.2 Gut microbiota modulation

Given the role of the gut–liver axis in NLRP3 activation, modulating the gut microbiota is a rational intervention direction. Approaches such as probiotics, prebiotics, and fecal microbiota transplantation can improve intestinal barrier function, reduce LPS translocation, and adjust the microbial metabolic profile, thereby attenuating, at the source, the gut-derived stimuli that provide Signal 1 for NLRP3. A randomized controlled trial demonstrated that allogeneic fecal microbiota transplantation improved abnormal small intestinal permeability in NAFLD patients, suggesting the feasibility of targeting the intestinal barrier (187). Although such strategies are relatively safe, their definitive efficacy in NAFLD still requires support from additional high-quality controlled studies.

#### 5.5.3 Blockade of downstream effectors and lifestyle intervention

At the downstream effector level, IL-1β neutralization strategies are supported by clinical data. A large-scale cardiovascular outcomes trial showed that IL-1β inhibition with the monoclonal antibody canakinumab reduced inflammation-related cardiovascular events without lowering blood lipids, confirming the clinical feasibility of blocking the IL-1β pathway (188); animal studies have also demonstrated that the IL-1 receptor antagonist anakinra attenuates NLRP3-driven hepatic inflammation and fibrosis (103), and IL-1 receptor blockade confers protection in inflammasome-dependent steatohepatitis (189). Together, these lines of evidence provide proof of concept for indirectly blocking the downstream effects of NLRP3. At the more terminal pyroptosis execution step, the FDA-approved drug disulfiram was found to inhibit pyroptosis by covalently modifying a key cysteine residue in GSDMD and blocking its membrane pore-forming activity, offering a candidate drug that can be repurposed to target the final step of pyroptosis (190). As a cautionary note, the pan-caspase inhibitor emricasan, although attenuating liver injury and fibrosis in mouse models of NASH (191), failed to improve liver histology and may even have worsened fibrosis and ballooning in a randomized controlled trial involving patients with NASH and fibrosis, suggesting that simple caspase blockade may divert cell death toward more inflammatory modalities (192). This negative result indicates that interventions targeting the downstream pathways of pyroptosis/apoptosis require careful evaluation, and the definitive value of the above downstream strategies in the hepatic indication of NAFLD remains to be fully validated. Finally, it must be emphasized that lifestyle intervention remains the cornerstone of NAFLD treatment: caloric restriction reduces lipotoxic signals and exercise improves the overall metabolic state, both of which can reduce the activation burden on NLRP3 from the upstream, and should be maintained throughout the course of any pharmacological intervention.

## 6 Conclusions and future directions

### 6.1 Core conclusions

Synthesizing the current evidence, the NLRP3 inflammasome is a central hub in the progression of NAFLD from simple steatosis to NASH, fibrosis, and ultimately hepatocellular carcinoma. Multiple metabolic danger signals—including lipotoxicity, oxidative stress and mitochondrial damage, endoplasmic reticulum stress, cholesterol crystals, and gut microbiota dysbiosis—can converge on common downstream nodes to drive NLRP3 activation, which in turn amplifies hepatic inflammation, promotes stellate cell activation, and drives fibrotic deposition through the maturation and secretion of IL-1β/IL-18 and GSDMD-mediated hepatocyte pyroptosis. Precisely because it occupies this central position in the pathological network, targeting the NLRP3 inflammasome is regarded as an important therapeutic direction with the potential to simultaneously intervene in inflammation and fibrosis.

### 6.2 Limitations of current research

Despite remarkable progress in mechanistic research, clinical translation remains significantly lagging. As a major public health challenge, NAFLD/MASLD urgently requires the translation of mechanistic discoveries into accessible interventions (193). First, the majority of evidence derives from animal models and *in vitro* experiments, and human studies—particularly large-scale, multicenter randomized controlled trials—are relatively scarce. There are also reports that pharmacological inhibition fails to confer benefit in certain models, suggesting that conclusions must be interpreted cautiously against the model background. In fact, in recent years, although multiple drugs for NASH have entered phase III clinical trials—for instance, the farnesoid X receptor agonist obeticholic acid achieved only limited fibrosis improvement from the FLINT to the REGENERATE trial (194, 195)—the overall efficacy has been modest, and some candidates, such as the ASK1 inhibitor selonsertib, failed to meet their primary endpoints (196). The CCR2/5 dual antagonist cenicriviroc also failed to sustain antifibrotic benefit in the final analysis of the CENTAUR phase 2b trial (197). At the same time, there has been positive progress, such as the pan-PPAR agonist lanifibranor meeting the composite endpoint of NASH resolution and fibrosis improvement in a phase 2b trial (198). These mixed experiences highlight the difficulty of therapeutic translation in MASLD/NASH and also provide complementary rationale for strategies targeting upstream inflammatory hubs such as NLRP3. Second, research on natural products and traditional Chinese medicine mostly remains at the preclinical stage, and the active constituents and mechanisms of action have not been fully elucidated. Third, the role of NLRP3 varies across different hepatic cell types, and in specific contexts such as liver cancer, it exhibits a dual pro-tumorigenic and anti-tumorigenic nature; simple systemic inhibition may lead to unintended consequences. In addition, much of the cell-type-resolved evidence—particularly for hepatic stellate cells and liver sinusoidal endothelial cells—derives from rodent or *in vitro* systems; hepatocyte- and Kupffer-cell/macrophage-based mechanisms are currently better supported in human tissue than stellate- or endothelial-cell-based ones, so the cell-type distinctions summarized in **Table 3** should be read as predominantly model-derived and not uncritically extrapolated to human MASLD. Furthermore, as an important component of innate immunity, the safety of long-term NLRP3 inhibition requires careful evaluation.

**Table 3:** Cell type-specific expression and function of the NLRP3 inflammasome in MASLD.

Cell type	NLRP3 expression	Main function	Pathological role in MASLD
Hepatocyte	Low at baseline, up-regulated under stress	Senses lipotoxicity/ER stress → pyroptosis → IL-1β/IL-18 release	Pyroptosis directly drives injury and inflammation amplification
Kupffer cell	High	Innate sensor → macrophage activation → cytokines	Drives hepatic inflammation and recruits peripheral immune cells
Hepatic stellate cell (HSC)	Up-regulated upon activation	Participates in fibrogenic signaling	Promotes HSC activation → myofibroblast → collagen deposition
Liver sinusoidal endothelial cell	Low	Maintains microenvironment homeostasis	Dysfunction under inflammation; promotes leukocyte adhesion
Infiltrating macrophage	High	Amplifies local inflammation	Aggravates MASH inflammation and fibrosis after recruitment

Evidence level: NLRP3 activity in hepatocytes and in Kupffer cells/infiltrating macrophages is the best supported and is documented at the cell-intrinsic level in human tissue; the entries for hepatic stellate cells and liver sinusoidal endothelial cells derive mainly from rodent and *in vitro* systems and describe the broader pathological function of these cells rather than firmly established cell-intrinsic NLRP3 activity in human MASLD.

### 6.3 Future research directions

At the basic research level, further elucidation is needed of the spatiotemporal activation patterns of the NLRP3 inflammasome at different stages of NAFLD, the functional differences of NLRP3 in hepatocytes, Kupffer cells, hepatic stellate cells, and liver sinusoidal endothelial cells, and the synergistic interactions and crosstalk with other inflammasomes such as NLRP1, NLRC4, and AIM2 (for example, AIM2 recognizes cytosolic double-stranded DNA and assembles a caspase-1-activating complex, and is closely linked to nucleic acid signals released during liver injury (199, 200); in hepatic ischemia-reperfusion injury, NLRP3 and AIM2 can be co-activated in Kupffer cells, suggesting a widespread role of NLRP3 in various liver injury settings (201). At the translational research level, efforts should be directed toward developing more specific and safer NLRP3 inhibitors, establishing biomarker-based patient stratification strategies, advancing the identification and structural optimization of active constituents from natural products, and exploring rational combination regimens of NLRP3 inhibitors with metabolic therapeutic agents. At the clinical research level, high-quality randomized controlled trials are needed to validate efficacy and safety, and, in the context of the updated MASLD nomenclature, the inclusion criteria and endpoint design should be re-evaluated, and the value of NLRP3-related indicators as biomarkers for disease progression and treatment response prediction should be explored.
